# Inter-study reproducibility of arterial spin labelling magnetic resonance imaging for measurement of renal perfusion in healthy volunteers at 3 Tesla

**DOI:** 10.1186/1471-2369-15-23

**Published:** 2014-01-31

**Authors:** Keith A Gillis, Christie McComb, John E Foster, Alison HM Taylor, Rajan K Patel, Scott TW Morris, Alan G Jardine, Markus P Schneider, Giles H Roditi, Christian Delles, Patrick B Mark

**Affiliations:** 1 Institute of Cardiovascular and Medical Sciences, British Heart Foundation Glasgow Cardiovascular Research Centre, 126 University Place, Glasgow, UK; 2 Glasgow Renal and Transplant Unit, Western Infirmary, Dumbarton Road, Glasgow, UK; 3 Department of Nephrology and Hypertension, University of Erlangen-Nuremberg, Erlangen, Germany; 4 Department of Radiology, Glasgow Royal Infirmary, Castle Street, Glasgow, UK

**Keywords:** Magnetic resonance imaging, Renal blood flow, Renal perfusion, Renal physiology, Arterial spin labelling

## Abstract

**Background:**

Measurement of renal perfusion is a crucial part of measuring kidney function. Arterial spin labelling magnetic resonance imaging (ASL MRI) is a non-invasive method of measuring renal perfusion using magnetised blood as endogenous contrast. We studied the reproducibility of ASL MRI in normal volunteers.

**Methods:**

ASL MRI was performed in healthy volunteers on 2 occasions using a 3.0 Tesla MRI scanner with flow-sensitive alternating inversion recovery (FAIR) perfusion preparation with a steady state free precession (True-FISP) pulse sequence. Kidney volume was measured from the scanned images. Routine serum and urine biochemistry were measured prior to MRI scanning.

**Results:**

12 volunteers were recruited yielding 24 kidneys, with a mean participant age of 44.1 ± 14.6 years, blood pressure of 136/82 mmHg and chronic kidney disease epidemiology formula estimated glomerular filtration rate (CKD EPI eGFR) of 98.3 ± 15.1 ml/min/1.73 m^2^. Mean kidney volumes measured using the ellipsoid formula and voxel count method were 123.5 ± 25.5 cm^3^, and 156.7 ± 28.9 cm^3^ respectively. Mean kidney perfusion was 229 ± 41 ml/min/100 g and mean cortical perfusion was 327 ± 63 ml/min/100 g, with no significant differences between ASL MRIs. Mean absolute kidney perfusion calculated from kidney volume measured during the scan was 373 ± 71 ml/min. Bland Altman plots were constructed of the cortical and whole kidney perfusion measurements made at ASL MRIs 1 and 2. These showed good agreement between measurements, with a random distribution of means plotted against differences observed. The intra class correlation for cortical perfusion was 0.85, whilst the within subject coefficient of variance was 9.2%. The intra class correlation for whole kidney perfusion was 0.86, whilst the within subject coefficient of variance was 7.1%.

**Conclusions:**

ASL MRI at 3.0 Tesla provides a repeatable method of measuring renal perfusion in healthy subjects without the need for administration of exogenous compounds. We have established normal values for renal perfusion using ASL MRI in a cohort of healthy volunteers.

## Background

Renal perfusion is a crucial component of normal renal function, being one of the main determinants of glomerular filtration rate and tissue oxygenation [1,2]. Serum creatinine and the derived estimated glomerular filtration rate (eGFR) are the conventional measures of renal function [3] used in clinical practice, however these are less sensitive to alterations in renal physiology. Furthermore, changes to these parameters may occur later in development of chronic kidney disease, or may be normal despite significant compromise in renal perfusion such as in the presence of renal artery stenosis. Measurement of renal blood flow may allow complementary assessment of renal haemodynamics and function; however this has been hindered in both research and clinical practice by the drawbacks of existing methods of measuring renal perfusion.

Clearance techniques have conventionally been used to measure effective renal blood flow, with para aminohippuric acid (PAH) clearance being the gold standard technique [4]. However, this process is labour intensive, time consuming, and invasive and inappropriate for use out with research studies. Furthermore, availability of PAH in the UK is limited due to debate as to whether it meets the legislative requirements regarding transmissible spongiform encephalopathy status of medical products for human use [5].

Dynamic perfusion studies performed using computed tomography (CT) or magnetic resonance (MR) imaging both require administration of an exogenous contrast compound which may be nephrotoxic, in the case of iodinated contrast used during CT examinations, which also carry an ionising radiation burden. Paramagnetic gadolinium based contrast agents for MRI, while generally safe, are inappropriate for use in renal impairment, due to concerns regarding an association with nephrogenic systemic fibrosis [6]. Nuclear scintigraphy requires exposure to ionising radiation as per CT scanning rendering it inappropriate for repeated use.

Arterial spin labelling magnetic resonance imaging (ASL MRI) is a novel technique which utilises magnetically labelled water protons in blood as an endogenous contrast agent, and as such represents a non invasive method of measuring renal perfusion without exposure to ionising radiation or exogenous contrast agents.

A number of ASL MRI sequences are available and have been reviewed previously [7]. Regardless of the ASL sequence, a number of scans must be taken, including the ASL contrast image, a background magnetisation image, and a T1 map. The T1 relaxation time reflects the duration of time taken for the magnetisation vector to recover to its baseline following a radiofrequency pulse. Different tissue types have different T1 values, with tissues with a greater proportion of water demonstrating longer values than fat or fibrosis.

Most perfusion MRI imaging in the literature is carried out at field strengths of 1.5 Tesla [8-11]. As magnetic labelling decays over the relaxation time T1, which is longer at higher field strengths, 3.0 Tesla MRI is associated with greater signal to noise ratio (SNR), which should result in enhanced image quality and allow more accurate analysis of renal perfusion. To this end, we investigated the reproducibility of ASL at 3.0 Tesla MRI in healthy volunteers with normal renal function.

## Methods

Healthy volunteers were recruited via advertisement. Subjects attended on three occasions; initially for screening questionnaire and blood and urine sampling, followed by ASL MRI undertaken during the second and third visits. Participants were fasted for 6 hours prior to imaging. Blood pressure was recorded on the day of study. All visits were completed within 4 – 28 days. All subjects gave written informed consent and the study was approved by the College of Medicine, Veterinary and Life Sciences University of Glasgow Ethics Committee.

### Arterial spin labelling magnetic resonance imaging

Magnetic resonance imaging (MRI) was performed on a Siemens Magnetom Verio 3.0 Tesla scanner (Siemens Erlangen, Germany), using a 6-channel phased array body coil. A localiser sequence was used to identify the location of the kidneys and the major vessels. ASL was performed using a flow-sensitive alternating inversion recovery (FAIR) perfusion preparation with a steady state free precession (True-FISP) pulse sequence. Five images with alternating selective and non-selective inversions were obtained in a single acquisition, and this was repeated five times. In addition, an image with no ASL preparation was acquired to allow the equilibrium magnetisation to be quantified. Sagittal oblique images were taken of both kidneys, with a single slice obtained at the midpoint of each axis, moved posteriorly to avoid major vessels. Fair True FISP parameters were: inversion time 900 ms, repetition time 3.65 ms, echo time 1.83 ms, flip angle 60°, field of view 380 mm by 380 mm, in plane resolution 256 × 256 and slice thickness 10 mm.

T1 maps were obtained during a separate breath hold using a modified Look-Locker inversion recovery (MOLLI) sequence.

### Image analysis

Renal morphology was assessed on the True-FISP localiser images using a commercially available multi modality post processing workstation (Siemens Syngo, Siemens Erlangen, Germany). Length, width and depth were measured and hence volume calculated using the ellipsoid formula (volume = length × width × depth × π/6) [12]. Volume was alternatively measured by tracing renal contours on each slice of a 22 slice transverse image, and multiplying the number of pixels within the region of interest, by the size per pixel and the slice thickness (the voxel count method). Kidney mass was then derived as a factor of kidney volume derived by voxel count, and the specific gravity of renal tissue, deemed to be 1.05 g/ml [13].

Image analysis was performed off line using bespoke MATLAB based software (MATLAB 2013, MathWorks, Natick, Massachusetts, U.S.A). Registration of the ASL images was performed using an enhanced correlation coefficient maximisation algorithm [14]. For each pair of selective/non-selective inversion images, the non-selective inversion image was subtracted from the selective inversion image. Finally, the average of the subtracted images was calculated. The differences in signal intensity between the selective and non-selective inversion images are small, and averaging over a number of subtractions improved the signal-to-noise ratio compared to a single subtraction. Pixels with intensity at the extremes of the range were excluded, as these were likely to represent adventitia or major vessels. Perfusion was determined on a pixel by pixel basis using the following formula [15]:

$$f=\frac{\lambda }{2\mathrm{TI}}\;\frac{\mathrm{\Delta M}\left(\mathrm{TI}\right)}{M_{0}}\;\exp\;\frac{\mathrm{TI}}{T1}$$

f is renal blood flow, λ represents the constant tissue-blood partition coefficient (0.8 mL/g), ΔM is subtracted difference of the selective and non-selective inversion images, at inversion time TI (900 ms). M_0_ is the equilibrium magnetisation and T1 is the measured longitudinal relaxation time at 3.0 T.

T1 relaxation time was measured at the cortex, medulla and for the whole kidney whilst perfusion values were derived for the cortex and the whole kidney. Absolute perfusion was calculated as a factor of kidney mass and whole kidney perfusion.

### Baseline biochemical measurements

Baseline serum biochemistry and haematology measurements and urinary protein and creatinine quantification were obtained at initial visit. Estimated glomerular filtration rate (eGFR) was calculated using the Chronic Kidney Disease Epidemiology Collaboration (CKD–EPI) formula [3].

### Statistics

Comparison of renal perfusion between right and left kidney, and ASL MRI 1 and 2, were made using paired Student’s t tests with p < 0.05 deemed to demonstrate significant differences between methods. Pearson correlation coefficients were used to determine correlation between MRI measurements, and between MRI measurements and serum and urine parameters. Bland Altman plots were made of the mean perfusion values against the difference between the values, with the 95% limits of agreement calculated as the mean difference plus or minus 1.96 times the standard deviation of the difference. Repeatability was also assessed using intra-class correlation (ICC), which measures the contribution of between subject variances to total variance. ICC lies between zero and one, with values closer to one indicating a stronger agreement between measurements. A two way random effect model was used with a 95% confidence interval. The within subject coefficient of variance (CV_ws_) is also expressed, which represents the ratio of the standard deviation of the differences between visits to the mean of all the perfusion measurements. Values closest to zero suggest good agreement between measurements made at each study. SPSS Statistics Version 19 was used for data analysis (IBM, Armonk, New York, U.S.A).

## Results

### Participant demographics

**Figure 1:**
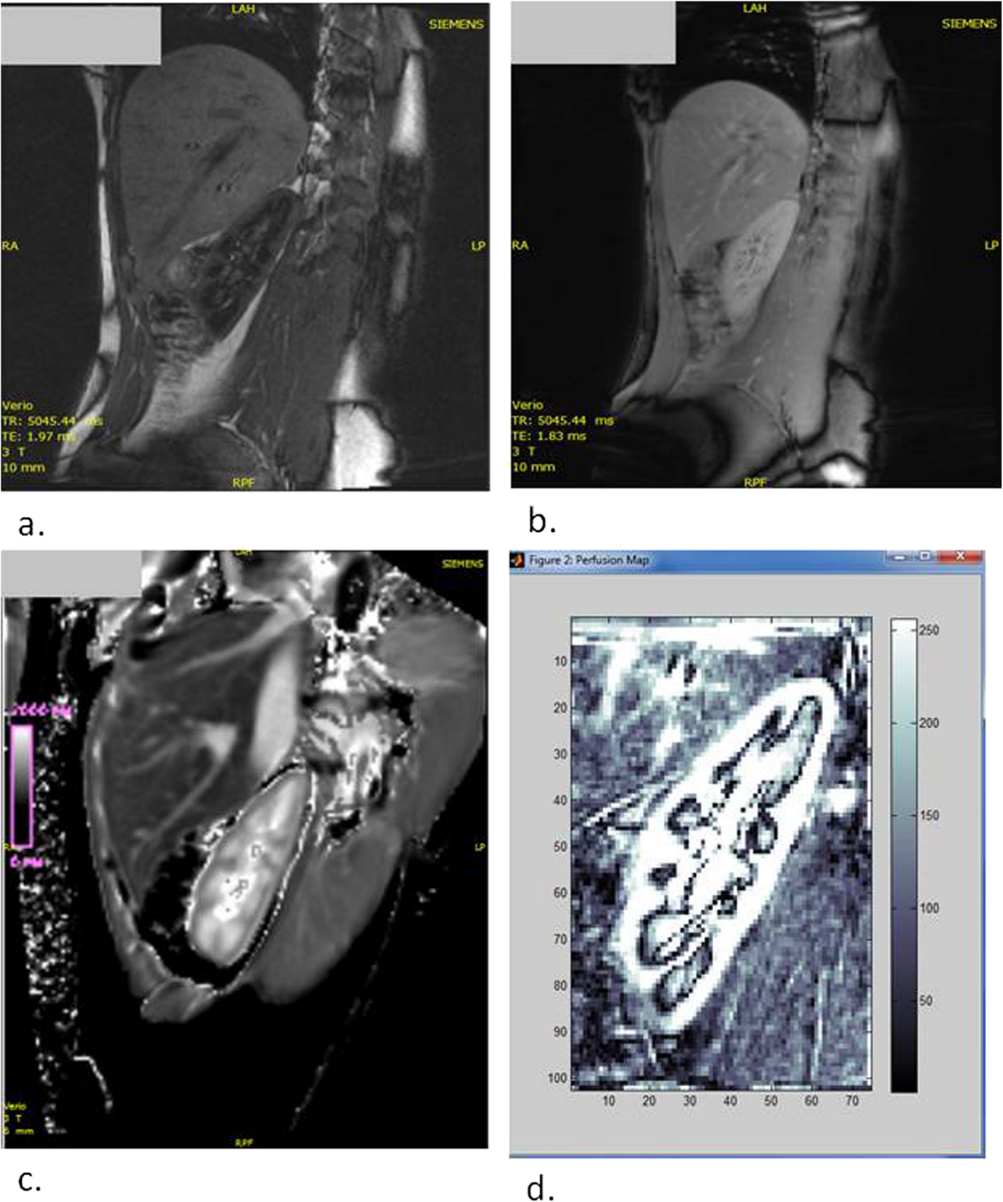
**Renal arterial spin labelling MRI images. a**. Single contrast ASL image. **b**. M0 image demonstrating magnetisation equilibrium **c**. T1 map. **d**. Composite image of 5 ASL MRI contrast images registered with post processing software.

12 participants completed the study protocol with a mean age of 44.1 ± 14.6 years. Mean blood pressure was 136/82 mmHg and no participants receiving antihypertensive therapy. All subjects had normal renal function with a mean CKD EPI eGFR of 98.3 ± 15.1 ml/min/1.73 m^2^ (Table 1) and no proteinuria was detected on laboratory quantification. Images of appropriate quality for analysis were obtained at both visits for all participants (Figure 1).

**Table 1:** Demographics and biochemistry of participating volunteers

	**Mean**	**SD**
Age *(years)*	44.1	14.6
Male *(number)*	5	
BMI *(kg/cm*^*2*^*)*	26.5	6.6
Systolic BP (*mmHg)*	136	23
Diastolic BP *(mmHg)*	82	9
Urea *(mmol/L)*	5.0	1.1
Creatinine *(μmol/L)*	72.3	10.6
CKD EPI eGFR *(ml/min/1.73 m*^*2*^*)*	98.3	15.1
Total Cholesterol *(mmol/L)*	4.8	0.7
HDL cholesterol *(mmol/L)*	1.8	1.2
Glucose *(mmol/L)*	5.2	1.0
Haemoglobin *(g/L)*	144	12
Protein to creatinine ratio *(mg/mmol)*	0.58	2.02
Albumin to creatinine ratio *(mg/mmol)*	0.17	0.32
Adjusted serum calcium *(mmol/L)*	2.47	0.05
Serum phosphate *(mmol/L)*	0.96	0.35
Calcium phosphate product *(mmol/L)*	2.38	0.89

### Renal morphology

**Figure 2:**
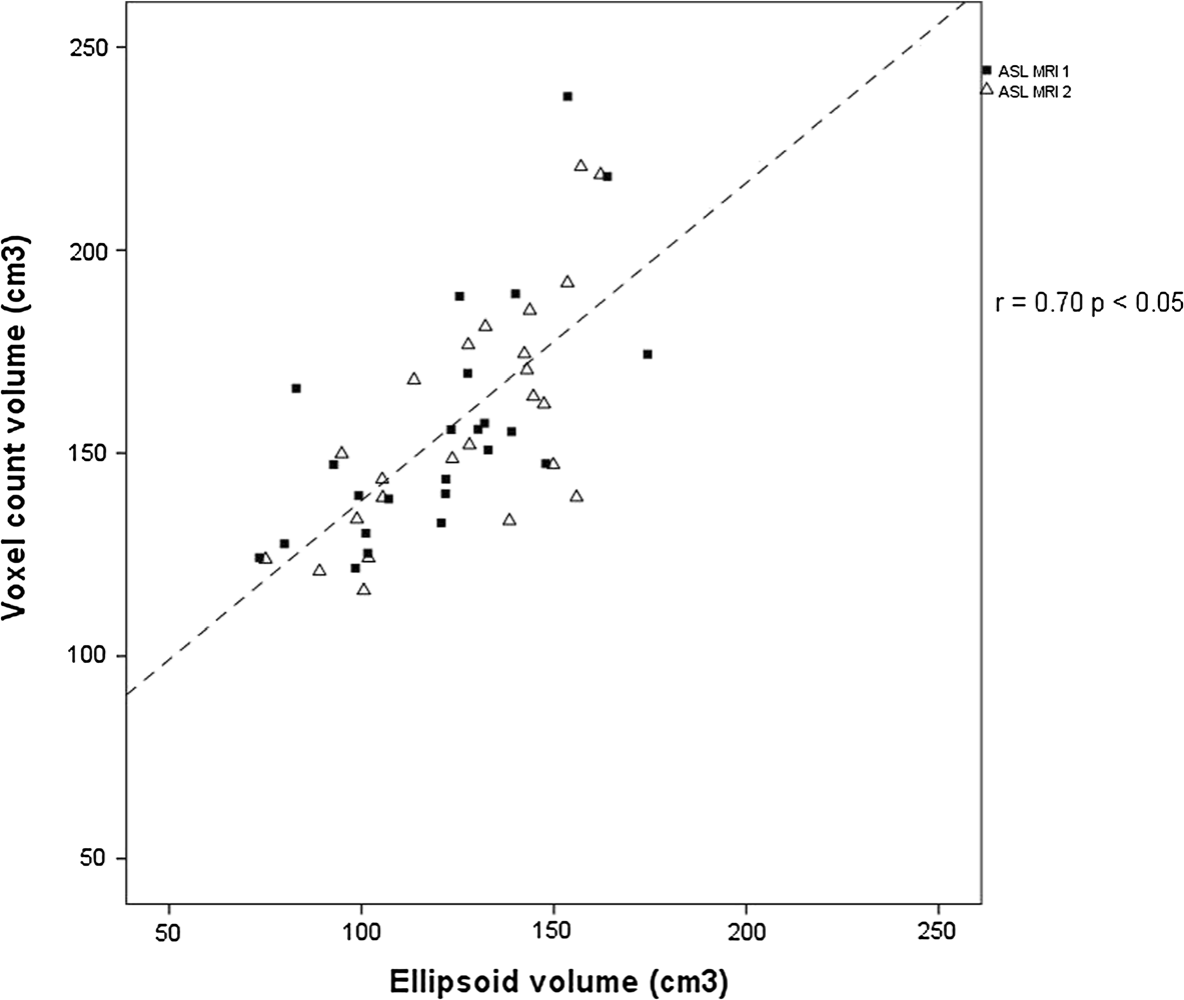
**Correlation plot of voxel count and ellipsoid formula methods of renal volume measurement using MRI.** R is the Pearson correlation coefficient.

Mean kidney length was 10.6 ± 0.8 cm at ASL MRI 1 and 10.8 ± 0.8 cm at ASL MRI 2 (Table 2) with significant correlation between the two (R = 0.89, p < 0.05). Kidney volume measured using the ellipsoid formula was 120.5 ± 26.1 cm^3^ at ASL MRI 1 and 126.4 ± 24.9 cm^3^ at ASL MRI 2. Kidney volume measured using the voxel count method was 155.7 ± 29.2 cm^3^ at ASL MRI 1 and 157.7 ± 28.6 cm^3^ at ASL MRI 2. Volume measurements made by the voxel count method were 30% higher than those made by the ellipsoid method, and there was significant correlation between both methods (R = 0.70, p <0.05) (Figure 2).

**Table 2:** Kidney morphological, T1 and perfusion measurements made by ASL MRI

	**ASL MRI 1**		**ASL MRI 2**		**p value**
	**Mean**	**SD**	**Mean**	**SD**	
Kidney length *(cm)*	10.6	0.8	10.8	0.8	0.02
Kidney volume Ellipsoid formula *(cm*^*3*^*)*	120.5	26.1	126.4	24.9	0.02
Kidney volume voxel count *(cm*^*3*^*)*	155.7	29.2	157.7	28.6	0.39
Whole kidney T1 *(ms)*	1491	61	1499	52	0.52
Cortical T1 *(ms)*	1376	104	1406	96	0.07
Medullary T1 *(ms)*	1651	86	1639	80	0.38
Whole kidney perfusion *(ml/min/100 g)*	228	40	230	41	0.66
Cortical perfusion *(ml/min/100 g)*	321	63	334	63	0.18
Absolute perfusion *(ml/min)*	367	66	379	86	0.33

Test of significance is Student’s t-test.

### Comparison of right and left kidneys

**Figure 3:**
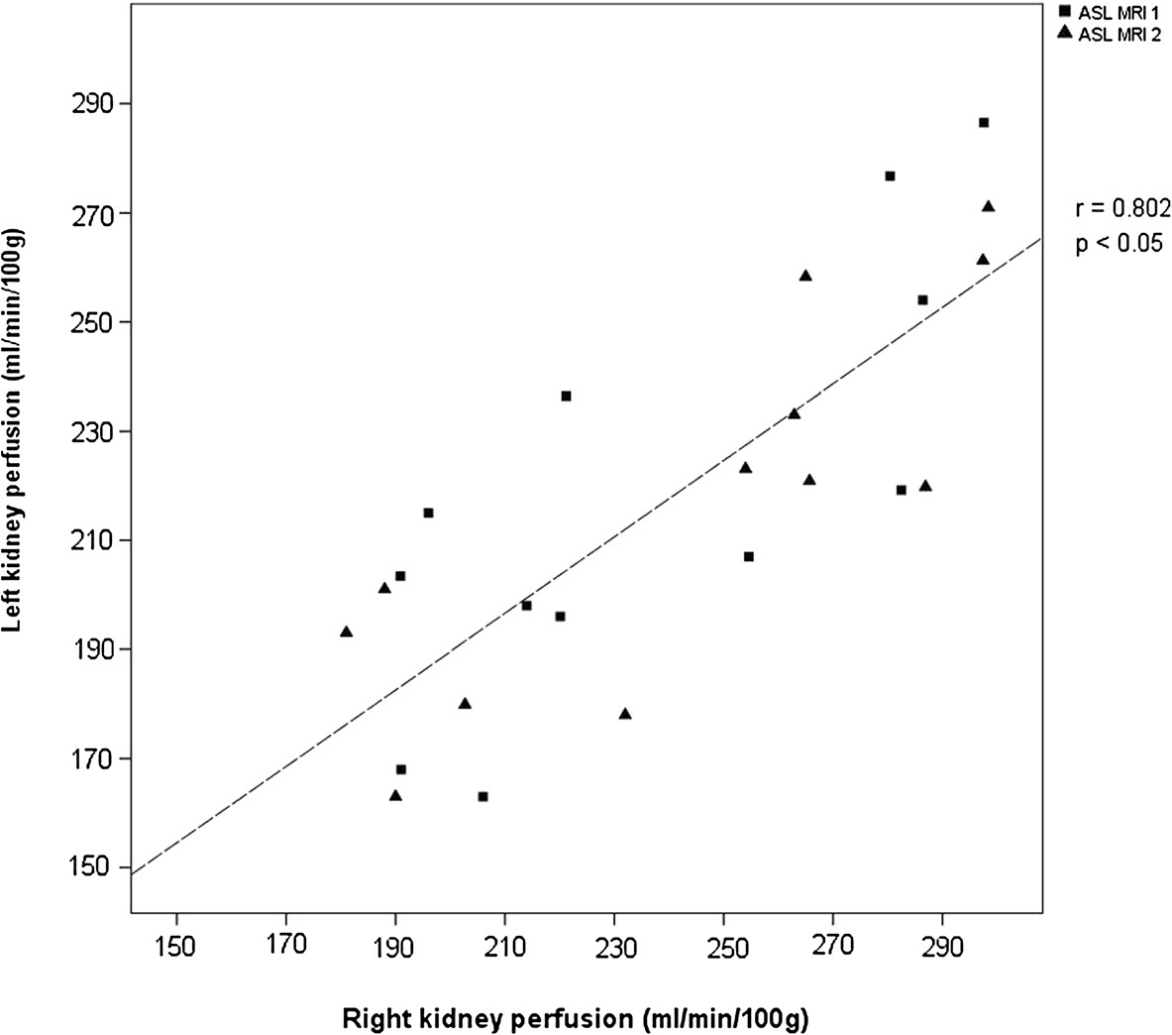
**Correlation of kidney perfusion measured in left and right kidneys.** R is the Pearson correlation coefficient.

Measurements of right and left kidneys were compared to exclude any confounding effect by differences in adjacent tissue types. No significant difference was observed in the T1 relaxation time of the cortex (p = 0.74), nor whole kidney (p = 0.56). Neither was there a difference in perfusion of the cortex (p = 0.93), or whole kidney (p = 0.28). Furthermore there was a significant correlation between the perfusion measured in the right and left kidneys of each participant between visits 1 and 2, both in the cortex (R = 0.79; p < 0.05), and the whole kidney (R = 0.80; p <0.05, Figure 3).

### T1 relaxation time

Mean T1 relaxation time was 1491 ± 61 ms at MRI 1 and 1499 ± 52 ms at MRI 2 (p = 0.52). Cortical T1 was 1376 ± 104 ms at MRI 1 and 1406 ± 96 ms at MRI 2 (p = 0.07), whilst T1 at the medulla was 1651 ± 86 ms at MRI 1 and 1639 ± 80 ms at MRI 2 (p = 0.38).

### Perfusion

**Figure 4:**
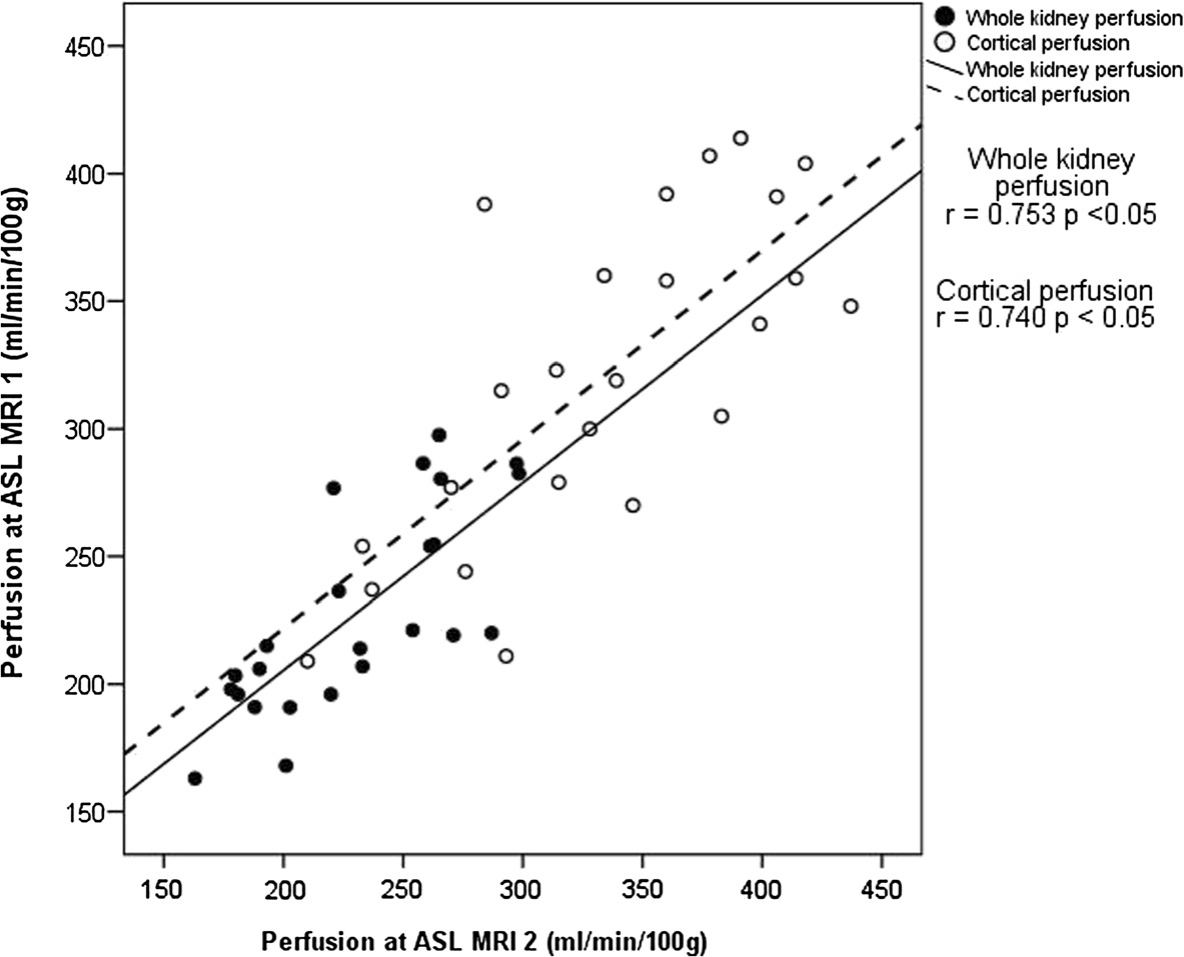
**Correlation between whole and cortical kidney perfusion measurements made at MRI 1 and 2.** R is the Pearson correlation coefficient.

Mean whole kidney perfusion was 228 ml/min/100 g on ASL MRI 1 and 230 ml/min/100 g on ASL MRI 2 (p = 0.66), with significant correlation between the two MRIs (R = 0.75, p < 0.05). Mean cortical perfusion was 321 ml/min/100 g then 334 ml/min/100 g (p = 0.18), with significant correlation between the two (R = 0.74, p <0.05) (Figure 4). Absolute kidney perfusion was 367 ± 66 ml/min at ASL MRI 1 and 379 ± 86 ml/min at ASL MRI 2 (p = 0.33), whilst total perfusion per participant was 734 ± 117 ml/min at ASL MRI 1 and 757 ± 156 ml/min at ASL MRI 2 (p = 0.42).

### Indices of repeatability

**Figure 5:**
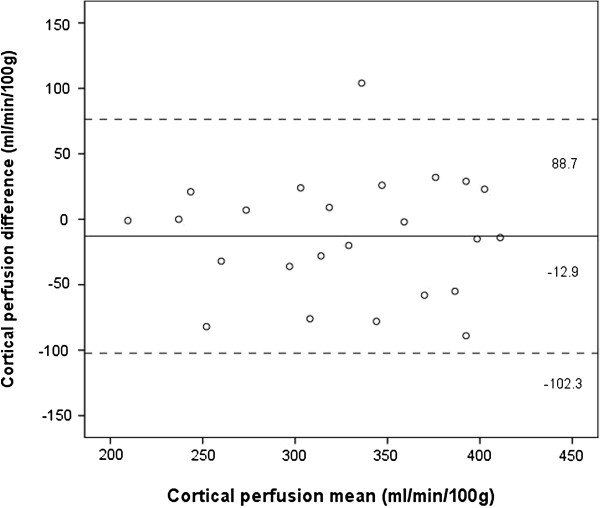
**Bland Altman plot of cortical perfusion measurements made at MRI 1 and 2.** Solid line and adjacent number indicates mean difference, whilst dashed line and number indicates limits of agreement.

**Figure 6:**
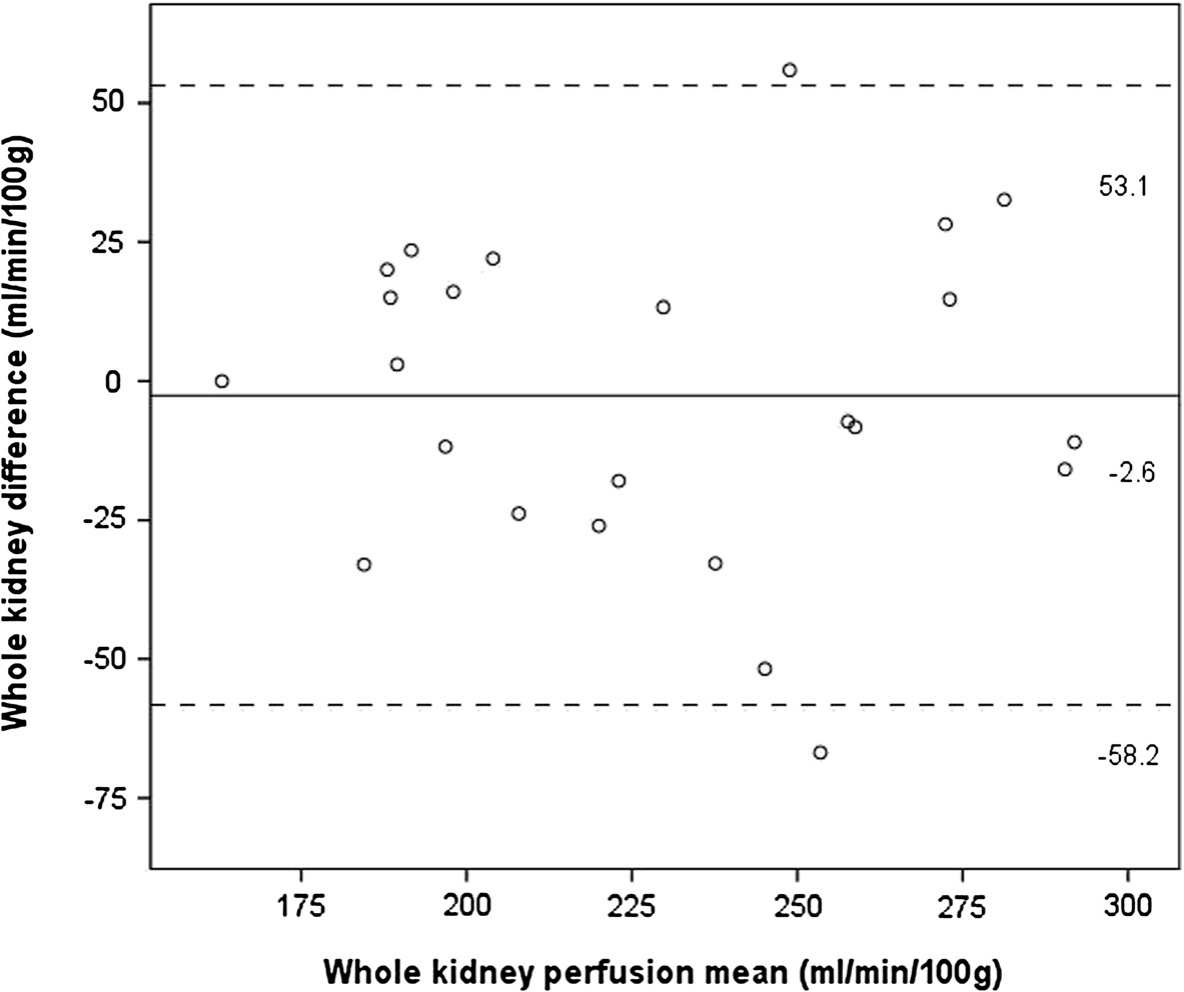
**Bland Altman plot of whole kidney perfusion measurements at MRI 1 and 2.** Solid line and adjacent number indicates mean difference, whilst dashed line and number indicates limits of agreement.

Bland Altman plots were constructed of the cortical and whole kidney perfusion measurements made at ASL MRIs 1 and 2 (Figures 5 and 6). These showed good agreement between measurements, with a random distribution of means plotted against differences observed. The intra-class correlation for cortical perfusion was 0.85 (95% confidence interval 0.65 – 0.94), whilst the CV_ws_ was 9.2%. The intra-class correlation for whole kidney perfusion was 0.86 (0.68 – 0.94), whilst the CV_ws_ was 7.1%.

### Correlation between physiological parameters and perfusion

No significant correlation was found between perfusion, and either blood pressure, serum creatinine, or CKD EPI eGFR.

## Discussion

Our study demonstrates the reproducibility of perfusion measurements made using FAIR True FISP ASL on a 3 T MRI in healthy volunteers, with no significant differences found between the first and second measurements made of either T1 relaxation time of renal tissue or renal perfusion.

T1 values were somewhat higher in our study than reported in the literature, at 1491 ms for the whole kidney, 1375 ms at the cortex, and 1651 at the medulla, at MRI 1. This is in comparison to reported values of 1142 ms at the cortex and 1545 at the medulla reported in one previous study [16], which used an inversion recovery method with different inversion times, in contrast to the multiple look locker sequence used here. MOLLI determination of T1 has advantages such as faster scan time, and smaller limits of agreement between repeated measures, than traditional T1 mapping techniques, and has been validated, at least in cardiac tissue [17,18].

Published data using ASL to measure renal perfusion in subjects with normal renal function vary widely with values quoted from 197 ml/min/100 g to 329 ml/min/100 g [11,19]. This disparity may represent physiological or population differences, however other factors pertaining to the ASL method are likely to also contribute to this. A number of ASL protocols have been employed [20], differing in breathing technique, field strength, motion correction, and with both single and multi slice approaches. Such lack of standardisation may hinder introduction of ASL MRI to routine clinical practice. Boss *et al.* previously showed the applicability of ASL at 3.0 T [21], and other studies have shown the reproducibility of FAIR ASL at lower field strengths [8,22]. Furthermore the FAIR ASL method employed in our study has, in earlier studies [23], been validated against the gold standard of a microsphere perfusion method of renal perfusion measurement. High correlation was found between FAIR ASL and microsphere perfusion in physiological conditions, and also after manipulation of perfusion with isoflurane and acetylcholine.

Our study provides further evidence for the reproducibility of FAIR ASL and confirms this at higher field strength than in previous work. To ensure uniformity of renal function and minimal variation in scan conditions in our cohort, subjects underwent biochemical screening of blood and urine and physical assessment to confirm normal kidney function prior to imaging. Furthermore, participants attended at a fixed time of day after a stated period of fasting to ensure constant study conditions. It is possible that differences in the ASL sequence, MRI systems or subject factors used in different studies may account for the variation in perfusion measurements.

Good reproducibility was demonstrated in the perfusion measurements made at 3.0 T MRI, with within subject coefficient of variation calculated at 9.2% for cortical perfusion, and 7.1% for whole kidney perfusion. This is similar to measures of reproducibility found in other studies at 1.5 T [8,22]. Therefore there was no demonstrable difference in the reproducibility of ASL measurements made at 1.5 T or 3.0 T in healthy volunteers. Signal to noise ratio is greater at higher field strengths, and whilst no advantage in terms of reproducibility was observed in our study, this may be of more benefit in the imaging of patients with chronic kidney disease where image quality may be compromised by patient factors such as obesity or breath holding ability, or tissue factors such as kidney fibrosis.

In addition to the numerous ASL sequences in use, different acquisition strategies are employed in order to minimise the error caused by respiratory motion. Consistent with our approach, other studies have employed breath-holding techniques, which whilst minimising respiratory motion, can prove difficult for participants to comply with. In our study all of our healthy volunteers were able to comply with a 25 second breath hold, however this strategy may not be appropriate for patients with kidney or co-morbid diseases. Other strategies which have been employed include prolonged acquisition during light free breathing, respiratory triggering, navigator echo and parallel imaging methods. Post-acquisition image realignment has been shown to improve visual quality and background noise suppression can be combined with parallel imaging to allow whole kidney data to be collected during free breathing [24]. The same study demonstrated that background suppression resulted in under estimated perfusion measurements, whilst motion correction led to improved estimation of perfusion [24]. Further research is required to improve the application of these techniques in order to give accurate quantification of renal perfusion.

Future work is required to refine renal perfusion measurements using ASL. Qualitative analysis of ASL images is possible using a model derived from the extended Bloch equation [15]. A limitation of this is that the model ignores transit time and exchange effects of water molecules in blood. A degree of the arterial labelling is lost from the point of the tagging site to the point of capillary exchange at the image slice by a factor relative to the transit time, and so the perfusion values determined by this can be complicated by this [25]. These limitations will have to be borne in mind during the use of ASL MRI in patients with chronic kidney disease given there may be significant alterations in transit time both between individuals and within individuals over time. Transit time could be measured as part of the ASL imaging protocol and perfusion values adjusted for this; further research is required to determine if this would be the appropriate approach.

We found no correlation between renal function and perfusion. This is mostly likely due to the high level of renal function present in this cohort, and one might expect to see a greater correlation when participants with a range of renal function are studied. Correlation between perfusion measured by ASL MRI and renal function measured by serum creatinine, or by invasive measurement of perfusion with para aminohippuric acid clearance has previously been demonstrated [10,11]. Perfusion maps generated via post processing result in a heterogeneous appearance of the renal medulla, probably due to the presence of larger vessels and the renal pelvis. This resulted in a degree of variability in the measurement of perfusion in this region, hence the measurement of whole kidney perfusion in preference to medullary perfusion. Improved post processing techniques may allow for differential quantification of cortical and medullary perfusion and measurement of these values may reveal differences in relative perfusion in patients with chronic kidney disease. Ideally automated detection of the differentiation between cortex and medulla using a digital threshold for signal intensity would generate more reliable, less operator dependent and hence reproducible data.

A further potential limitation is that the True-FISP localiser images from which renal morphological data is obtained provide only a limited number of slices of limited anatomical quality, and sequence development is likely to lead to a more accurate appraisal of kidney size in the future. We found that the two methods of volume measurement correlated but with 30% larger measurements found using the voxel count method, as in earlier studies [12].

There is currently no *in vivo* measure of renal perfusion, which can be performed non-invasively or without exposure to exogenous compunds or ionising radiation in patients with significant renal impairment. Renal perfusion can be measured invasively in animals using microsphere techniques [23], and this has proved useful to validate ASL MRI perfusion measurements against a gold standard. PAH clearance is the gold standard method of assessing renal plasma flow in humans, from which the renal blood flow can be determined by a scaling factor dependent on the haematocrit. The technique is time consuming for participants however, with a protocol lasting up to two hours [4]. It also requires intravenous cannulation and can be associated with complications such as anaphylaxis. Typical PAH clearance measurements are of the order of 500 – 600 ml/min. However due to incomplete renal excretion of PAH, the technique underestimates plasma flow by around 10-20%, and to a greater extent when plasma flow is less than 300 ml/min [26].

These limitations have led to application of ASL being used for assessment of renal perfusion in a small number of clinical studies. Whilst total scan time is dependent on the sequence and protocol, the duration is of the order of 15 – 25 minutes and therefore provides for the possibility of dynamic measurements of renal perfusion, following therapeutic intervention. This provides an inherent advantage over both PAH and gadolinium contrast enhanced MRI, which are not repeatable over such a short time frame. Whilst image analysis can be time consuming, the short scan time entailed by ASL MRI is of benefit to recruitment and retention during clinical studies. Image analysis time in our study was approximately 30 minutes per kidney, including renal volume, T1 and all ASL measurements. One study has demonstrated a significant increase in renal perfusion quantified with ASL, using the renin inhibitor aliskiren [27]. The same group have shown renal denervation for resistant hypertension does not appear to impact on renal perfusion, again assessed with ASL MRI [28]. The implications of these studies suggest that ASL MRI represents a novel method for testing the renal haemodynamic consequences of therapeutic interventions, without resorting to time consuming methods entailing administration of exogenous materials, or the use or ionising radiation.

Therefore MRI ASL may potentially provide useful insights into the pathophysiology of a number of conditions where renal perfusion is altered, including acute kidney injury, heart failure and renal arterial disease [29-31], or have clinical utility across a broad spectrum of chronic kidney disease, including renal transplantation [10]. Furthermore, ASL MRI appears to have a role in differentiating histological subtypes of renal masses, and may in time form part of a ‘one stop shop’ imaging platform for assessment of renal tissue in health in disease [32]. MRI based studies will always be limited by the relatively high cost of imaging, more limited access to scanners compared to ultrasound and the relative inconvenience and tolerability of the MRI examination for patients. Finally, by combining our work with other studies, reference ranges for renal perfusion with ASL MRI can be established [8,19].

## Conclusion

In summary, development of renal ASL MRI represents a technique, which may be applicable, both for diagnostic purposes and for monitoring response to therapeutic interventions. We have demonstrated ASL MRI to be reproducible at in healthy volunteers with normal kidney function at 3.0 Tesla. Development of widely available MRI sequences and software analysis platforms will permit more widespread use of ASL MRI in clinical practice. Further research is required to investigate its utility across a spectrum of renal disease.

## Competing interests

We have no competing interests to declare.

## Authors’ contributions

KAG recruited the subjects, analysed MRI studies and drafted the manuscript. CM, JEF, GHR performed MRI analysis. AHMT, RKP recruited subjects and refined MRI analysis. STWM, AGJ, participated in the design of the study and drafted the manuscript. MPS, CD, PBM conceived the study, and participated in its design and coordination and helped to draft the manuscript. All authors read and approved the final manuscript.

## Pre-publication history

The pre-publication history for this paper can be accessed here:

http://www.biomedcentral.com/1471-2369/15/23/prepub
